# Entropy of Conduction Electrons from Transport Experiments

**DOI:** 10.3390/e22020244

**Published:** 2020-02-21

**Authors:** Nicolás Pérez, Constantin Wolf, Alexander Kunzmann, Jens Freudenberger, Maria Krautz, Bruno Weise, Kornelius Nielsch, Gabi Schierning

**Affiliations:** 1 Institute for Metallic Materials, IFW-Dresden, 01069 Dresden, Germany; 2 Institute of Materials Science, TU Dresden, 01062 Dresden, Germany; 3 Institute of Materials Science, TU Bergakademie Freiberg, 09599 Freiberg, Germany; 4 Institute for Complex Materials, IFW-Dresden, 01069 Dresden, Germany; 5 Institute of Applied Physics, TU Dresden, 01062 Dresden, Germany

**Keywords:** electronic entropy, Seebeck coefficient, transport, LaFeSi, FeRh, CuNi

## Abstract

The entropy of conduction electrons was evaluated utilizing the thermodynamic definition of the Seebeck coefficient as a tool. This analysis was applied to two different kinds of scientific questions that can—if at all—be only partially addressed by other methods. These are the field-dependence of meta-magnetic phase transitions and the electronic structure in strongly disordered materials, such as alloys. We showed that the electronic entropy change in meta-magnetic transitions is not constant with the applied magnetic field, as is usually assumed. Furthermore, we traced the evolution of the electronic entropy with respect to the chemical composition of an alloy series. Insights about the strength and kind of interactions appearing in the exemplary materials can be identified in the experiments.

## 1. Introduction

Entropy provides information about the degrees of freedom or ordering of a statistical collectivity, i.e., it is macroscopically seen and treated as an entity. This order directly correlates with changes in the density of states of the respective statistical collectivity. For electrons in crystalline solids, this information is usually extracted from band structure theory assumptions. It is valid in the case that the sometimes quite stringent assumptions of the theoretical model are met. Experimental systems inherently deviate from the ideal solid state model. Due to this, the density of states calculated theoretically is sometimes not enough to describe the electronic properties in real systems. Typical cases where changes in the electronic density of states occur are charge order/disorder phenomena, such as the formation of charge density waves phases, superconducting phases, Fermi liquid systems, or other correlated electron systems. Further systems that are challenging to describe by theoretical solid state considerations are disordered solids, such as alloys, amorphous materials, materials with complex elementary cells, or materials containing a high number of defects induced, for instance, by the fabrication technology.

A usual approach to evaluate the total electronic entropy *S_E_* of a crystalline solid from experimental data is to analyse the low temperature specific heat capacity, *c_p_*, measurements under the assumption of a free electron gas [1]. Here the Sommerfeld coefficient is the relevant value, experimentally obtained by fitting the low-temperature *c_p_* data. While this is currently the most widely applied method for an *S_E_* characterization of crystalline solids, there are some intrinsic drawbacks to this method. These come on the one hand from the assumption of a free electron gas and on the other hand from the fact that the relevant materials properties can only be inspected at low temperature [1]. Both rule out the investigation *S_E_* changes at phase transition, especially those occurring at temperatures above 20 K, and such that induce electronic ordering phenomena. 

Within this article, we discuss a recently suggested method for the *S_E_* characterization [2] that overcomes some of the limitations of the low temperature *c_p_* analysis, providing a tool for investigating such mentioned electronic systems by a direct experimental approach. We herein utilize the thermodynamic description of the Seebeck coefficient, *α*, originally described by Onsager [3,4], and later referred to by Ioffe [5] in order to describe the *S_E_* of solids. The inherent advantage of the thermodynamic interpretation of *α* is that it is not bound to any model, provided the statistical description of the system is significant. 

The idea to measure the *S_E_* through the measurement of macroscopic electronic properties like the Seebeck of Thomson effect has been discussed in literature [4,5,6,7,8], and dates, in principle, back to Thomson (Lord Kelvin) who interpreted that the Thomson effect could be seen as the specific heat of electrons, whereas the Seebeck coefficient would be the electronic entropy (divided by the charge of the electrons) [9]. Rockwood [9] pointed out that the measurement of thermoelectric transport properties necessarily only addresses the electrons that participate in the transport. He therefore specified the term “electronic transport entropy” to distinguish from a “static electronic entropy”. Furthermore, thermoelectric transport measurement could never be done under truly reversible conditions since the sample needs to be exposed to a temperature gradient and is therefore not under isothermal conditions. Still, he came to the conclusion that the measurement of the thermoelectric coefficients would most likely provide the only practical and generally valid method by which partial molar entropies of electrons could be obtained. Peterson and Shastry construed the Seebeck coefficient as particle number derivative of the entropy at constant volume and constant temperature [8]. Despite this given theoretical framework, examples in which Seebeck coefficient measurements were used to quantitatively deduce *S_E_* are rare and recent but still prove the broad applicability. Our group showed that *S_E_* of a magneto-caloric phase transition could be obtained by thermoelectric transport characterization [2]. Small entities of particles like quantum dots can likewise be characterized [10]. At high temperatures, molten semiconductors and metals were similarly studied [11]. Within this paper, we will discuss the broad applicability of this method. For the following discussion, we refer to the description of the electronic entropy per particle, *S_N_*, as derived within a recent review, providing an applied view on the thermodynamic interpretation of *α* [12]:

*S_N_* = *α* · *e* (1)

where *e* is the charge of the particle.

In simple metals, a formal expression of α can be derived from band structure arguments as in the case of the Mott formula [13]. Often, a single parabolic band model is assumed. Herein, the relation between α and the density of states becomes evident, thus establishing a direct connection between α and *S_E_*. While the general thermodynamic interpretation of α does not rely on any kind of model, the Mott formula already contains simplifications and assumptions. From the description of the quantity *S_N_* as introduced in Equation (1), it is suggested that there exists an absolute value of *S_N_* since α is a quantity that also has an experimentally accessible defined zero-level rather than a relative one where only changes in the quantity can be considered. The case of *α* = 0 occurs, for example, (i) in the superconducting state of matter, where electrons all condense at the state of lowest energy possible and therefore per definition a situation of zero entropy [14] and (ii) in the compensated case that electrons and holes exactly transport the same amount of heat, i.e., intrinsic semiconductors have zero Seebeck coefficients [15]. The latter is an often-seen zero crossing of an n-type conductivity mechanism to a p-type conductivity mechanism. Then, the measured α *= 0* corresponds to the overall observable α of the material. Naturally, the contributions of the individual bands contain electronic entropy contributions with *S_E_*, _individual subband_ *≠ 0*. The full evaluation of *S_E_* from α requires a correct description of the collectivity of electrons in the system. This is the point in the complete line of argumentation where assumptions and simplifications necessarily enter the picture. In order to experimentally obtain the entropy of the entity of electrons that participate in the transport, referred to as electronic entropy, *S_E_*, the number of electrons contributing to the Seebeck voltage needs to be known. In principle, any experimental procedure to obtain the charge carrier density, *n*, could be used. Herein, it is, as, for instance, suggested in [2,11]:

*S_E_* = *n* · *S_N_* = *n* · *α* · *e* (2)

In this work, we measure the ordinary Hall coefficient *R_H_* to obtain *n*, using the relation *R_H_ = 1/(n·e)*. By doing so, we introduce the strong assumption of a parabolic single-band transport model that is inherent to any Hall measurement. Combining both quantities, we can give a measure of *S_E_*:

*S_E_* = α/*R_H_* (3)

We present examples that highlight the relevance of the entropy interpretation of α and provide insight into the electronic properties: (1) magneto-structural phase transitions of an intermetallic Ni-doped iron rhodium phase, Fe_0.96_Ni_0.02_Rh_1.02_ (FeRh) [16], and an intermetallic lanthanum iron silicon phase, LaFe_11.2_Si_1.8_ (LaFeSi) [17,18,19,20,21]; (2) alloying in the copper–nickel (CuNi) solid solution series.

## 2. Materials and Methods 

All samples characterized within this work were obtained by arc melting, and followed by specific temperature treatments to ensure a homogenous microstructure. Details about the fabrication and structural characterization of the samples can be found in [22] and in [23] for LaFe_11.2_Si_1.8_ (LaFeSi). The samples investigated in the present paper stem from the same batches as the indicated references. In the case of the CuNi alloy series, the processing followed a combination of homogenization (973 K, 5 h) with quenching in H_2_O, hot rolling (1173 K) and recrystallization (973 K, 1 h).

The transport characterization was performed depending on the temperature range using physical property measurement systems of the Quantum Design DynaCool series and the Versalab series using the thermal transport option for α and the electrical transport option for the Hall characterization in standard Hall bar geometry [24]. For the CuNi alloy series, a Linseis LSR 3 device was used to measure the near-room temperature α (315 K) and electrical conductivity, *σ*.

The microstructure of the samples was routinely investigated by scanning electron microscopy and X-ray diffraction.

## 3. Results and Discussion

As briefly discussed above, the entity of carriers needs to be known for the statistical interpretation of α. Following Equation (2), we utilize *n* obtained from a Hall-effect measurement. Herein, one has to be aware of the fact that this evaluation method may be affected by multi-channel transport, induced by multiple bands. However, given a minimal set of regularities, we can compare a homogenous series of samples or one sample under different experimental conditions consistently. 

### 3.1. Magneto-Structural Phase Transition

The first example is related to meta-magnetic phase transitions in two magneto-caloric materials, namely Ni-doped FeRh and LaFeSi. They represent examples for a system that can be described with a band magnetism model (FeRh) [25] and a system with a component of localized ionic magnetism (LaFeSi) [26]. General information on the total entropy change in the phase transition of FeRh can be found in Ref. [2] and references therein, as well as a discussion of *S_E_* of this phase transition derived by transport measurements. Additionally, LaFeSi is a well-studied material with respect to magnetic and lattice entropy [26,27,28]. Due to soft phonon states close to transition, the lattice entropy change is large [29], but a combined contribution of lattice entropy and *S_E_* was suggested [27]. Details on the transport properties of LaFeSi are given in literature with respect to α [28,29] and the anomalous Hall effect [30].

**Figure 1:**
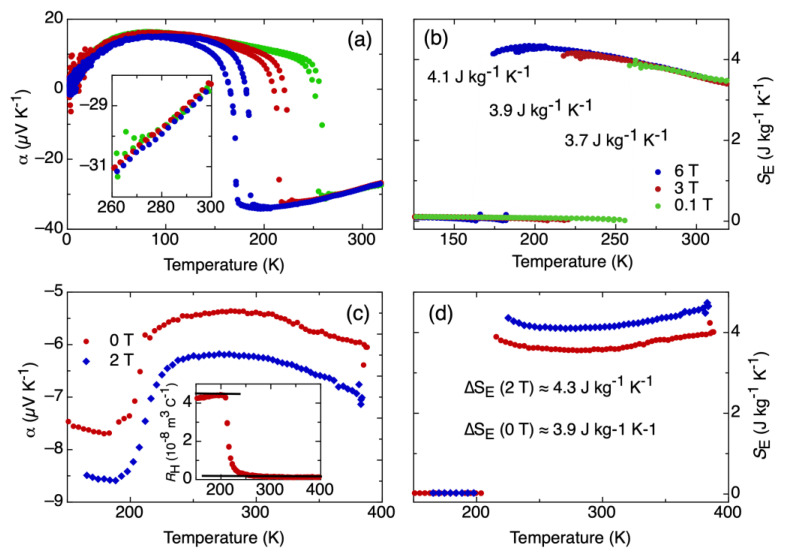
Seebeck coefficient and entropy evaluation in Ni-doped FeRh (**a**,**b**) and LaFeSi (**c**,**d**). Inset to (**a**): enlarged view of the high temperature region. Inset to (**c**): Measured Hall coefficient of LaFeSi.

The impact of the applied magnetic field on the transport of the mobile charge carriers shows a clear distinct signature in both materials, which we will discuss in the following. Both magnetic systems behave differently, as best seen in α. In the case of FeRh (Figure 1a), it can be seen that the temperature of the phase transition depends on the magnetic field. This is a striking difference of the *S_E_* evaluation by transport experiments and calorimetric measurements that—for intrinsic reasons—do not allow this difference to be unveiled. The *α* far from the phase transition is independent of the strength of the magnetic field, as emphasized in the inset in Figure 1a that shows an enlarged view of the data in the main panel. In contrast, in the case of LaFeSi (Figure 1c), the α far from the phase transition shows a clear difference in the value depending on the magnetic field. Interestingly, the magnitude of α increases as a magnetic field is applied. The inset to Figure 1c shows the measured Hall coefficient, and the black lines indicate the levels used for the entropy evaluation as was similarly done in [2]. We get a value corresponding to the *∆S_E_* at the phase transition, as depicted in Figure 1b,d. In both cases, we see *∆S_E_* of a comparable magnitude around 4 J K^−1^ kg^−1^. Moreover, the absolute values of the obtained *S_E_* are also comparable. Furthermore, in both cases, an increase of *∆S_E_* is observed when a magnetic field is applied. However, the apparent origin of the increase in *∆S_E_* for both materials is different. In the case of FeRh, the first order meta-magnetic transition shifts to lower temperatures as the field is applied (Figure 1a,b). Accordingly, *α* follows a monotonic trend until the phase transition occurs. In the case of the LaFeSi, the amount of Si (x = 1.8) is on the threshold for changing the transition type to the second order [28]. Therefore, the transition temperature does not shift significantly, and only a slight broadening is observed. In this case, it is the change of the over-all entropy level with the applied magnetic field (Figure 1c,d) that causes the increase in *∆S_E_*. In the case of LaFeSi, this could be an indication of the interaction between itinerant electrons and localized moments, causing the increase of *S_E_* with magnetic field. There is no such interaction in the FeRh case, as magnetism resides to a dominant part within the conduction electrons. Besides minor numerical corrections to the presented results (compare discussion Ref. [2]), it is clear that this method of analysis provides an insight to the interactions relevant to the conduction electrons that go beyond what typical calorimetric experiments can offer. 

### 3.2. Alloying

**Figure 2:**
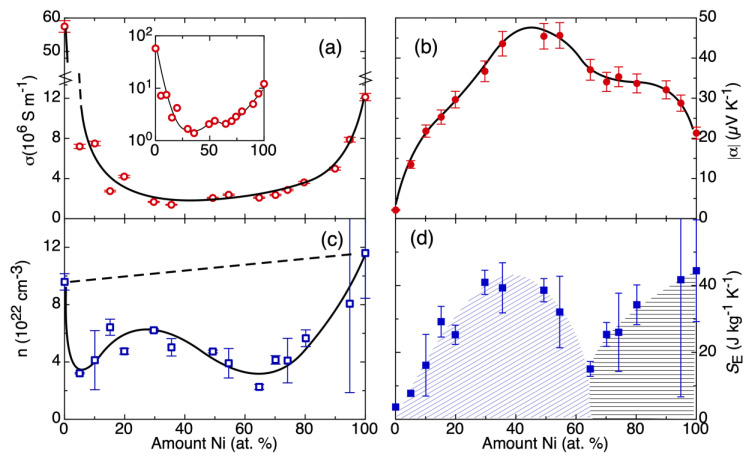
Thermoelectric and transport properties across alloy system Cu–Ni at room temperature, alloy composition was obtained with Energy-Dispersive X-Ray spectroscopy: (**a**) electrical conductivity, (**b**) the Seebeck coefficient in absolute values, (**c**) the carrier concentration derived from the Hall coefficient, (**d**) calculated electronic entropy. Lines and shades are guides to the eye.

The differential evaluation of a systematic series of homogenized CuNi alloys with respect to their |α|, σ, *n*, and *S_E_* at room temperature is shown in Figure 2. Herein, it is the specific situation of alloys that they typically cannot be accurately calculated or predicted by usual band structure models. However, the full alloy series is experimentally accessible. There are no structural phase transitions reported, and, also, all investigated samples were homogenous with respect to their microstructure and composition by scanning electron microscopy and X-ray diffraction. The dependence of σ on the Ni content (Figure 2a) presents two minima at around 30 at.%-Ni and at around 70 at.%-Ni, which are better seen in the inset to Figure 2a, where the data of the main panel are presented in logarithmic vertical scale.

In the trend of |α| (Figure 2b), a broad maximum can be seen slightly below to the equiatomic composition, close to the composition of the highest chemical disorder, a similar situation to that of other entropic parameters of such alloys [31], but a shoulder at a composition of about 70 at.%-Ni is also evident. This observation of high |α|for a high chemical disorder reflects the general finding that high configurational entropy is a prerequisite for the observation of large |α| [32]. Because of the close relationship between large |α| and high configurational entropy, it was recently suggested to even use configurational entropy as a gene-like performance indicator for the computational search of new thermoelectric materials [33].

The parameters σ and |α| follow inverse trends with respect to one another. Additionally, these trends match the description of *α* under the Mott formula [13]. Consequently, the investigated alloy series represents a good electronic model system. There is no clear trend in the data of *n*. (Figure 2c) The pure metals Cu and Ni have the highest *n*. Different effects superimpose to a more sophisticated dependence of *n* on the alloy composition: (i) the effect of change in the average lattice parameter by the alloying [31] should create a gradual increase in *n* as the amount of Nickel increases; (ii) additionally, with the addition of Ni (Ni: 3d^8^ 4s^2^; 2 electrons per Ni atom) into the Cu matrix (Cu: 3d^10^ 4s^1^; 1 electron per Cu atom) more charge will also be added [34]. A linear increase is schematically depicted by the dashed line in Figure 2c. The overall result of these measurements is a clear minimum at approximately 65 at.%-Ni. This already indicates that additional degrees of complexity add to this simplified picture.

The combination of |α| and *n* to extract the *S_E_* allows us to gain additional information compared to the individual transport coefficients. Figure 2d shows a curve in *S_E_* with maximum at approximately 30% of Ni and an additional clear minimum at approximately 65% of Ni. Coming from the Cu-side of the phase diagram, the increase in *S_E_* points out an increase in the available states for the transport electrons, which may be intuitively understood: the disorder in the non-periodic electrostatic potential leads to an increase in the entropy of the transport electrons. This increase in *S_E_* reaches a maximum close to the point where the maximum chemical disorder is expected, following the trend of |α|. Coming from the Ni-side of the phase diagram, |α| increases and *n* decreases. The |α|, similar to the Cu-side of the phase diagram, shows higher values because of a higher degree of chemical disorder in the system. But the |α| does not follow a monotonic trend; instead, it has a plateau. This, combined with the reduction of *n* in the same composition region, results in a sharp minimum of *S_E_*. This minimum exactly coincides with the onset of ferromagnetism in the alloy series. Hence, the entropy evaluation provides an insight on how the magnetic ordering mechanism in this alloy affects the localization of charges, possibly due to interactions between d- and s-orbitals. While there is no one-to-one correspondence between the experiment and the microscopic origin, it still provides a meaningful measure of the intensity of correlations in the electronic transport system, which are not easily accessible by usual ab-initio methods. 

## 4. Conclusions

In conclusion, this proposed method provides a good instrument for the characterization of electronic interactions or correlations in the material, although the absolute values of *S_E_* or *∆S_E_* obtained may, in some cases, need to be corrected (further discussed in [1]). In the case of magnetocaloric materials, the effect of the magnetic field on the electronic entropy change can be traced. In the case of alloys, the effect of the atomic disorder can also be traced on the free electrons. In order to gain deeper insight on the physics of disordered systems or systems with concurring interactions, the goal of future research might be to develop the statistical methods under the point of view of thermodynamics that would allow us to describe the statistical collectivity of electrons. In this way, we could transform the qualitative results of our experiments into quantitative predictions.
